# Sarcopenia, obesity, and natural killer cell immune senescence in aging: Altered cytokine levels as a common mechanism

**DOI:** 10.18632/aging.100482

**Published:** 2012-08-29

**Authors:** Charles T. Lutz, LeBris S. Quinn

**Affiliations:** ^1^ Department of Pathology and Laboratory Medicine, Department of Microbiology, Immunology, and Molecular Genetics, and the Markey Cancer Center, University of Kentucky, Lexington, KY 40536, USA; ^2^ Geriatric Research, Education, and Clinical Center, VA Puget Sound Health Care System, Seattle, WA 98108, Division of Gerontology and Geriatric Medicine, Department of Medicine, University of Washington, Seattle, WA 98195, and Seattle Institute for Biomedical and Clinical Research, Seattle, WA 98108, USA

**Keywords:** Skeletal muscle, adipose tissue, Sarcopenia, obesity, immunity, natural killer lymphocytes, aging

## Abstract

Human aging is characterized by both physical and physiological frailty. A key feature of frailty, sarcopenia is the age-associated decline in skeletal muscle mass, strength, and endurance that characterize even the healthy elderly. Increases in adiposity, particularly in visceral adipose tissue, are almost universal in aging individuals and can contribute to sarcopenia and insulin resistance by increasing levels of inflammatory cytokines known collectively as adipokines. Aging also is associated with declines in adaptive and innate immunity, known as immune senescence, which are risk factors for cancer and all-cause mortality. The cytokine interleukin-15 (IL-15) is highly expressed in skeletal muscle tissue and declines in aging rodent models. IL-15 inhibits fat deposition and insulin resistance, is anabolic for skeletal muscle in certain situations, and is required for the development and survival of natural killer (NK) lymphocytes. We review the effect that adipokines and myokines have on NK cells, with special emphasis on IL-15. We posit that increased adipokine and decreased IL-15 levels during aging constitute a common mechanism for sarcopenia, obesity, and immune senescence.

## INTRODUCTION AND PRELIMINARY OBSERVATIONS

An inevitable consequence of human and rodent aging is sarcopenia--loss of muscle mass [1]. Some muscle loss is due to physical inactivity, but even highly trained athletes lose muscle mass and strength with age [2-6]. Although exercise programs can prevent and/or ameliorate sarcopenia, the effectiveness of exercise interventions to build muscle and effect metabolic improvements is less efficient in elderly subjects than in the young, due to multiple cellular and biochemical changes [7, 8]. Frailty is a clinically-defined condition that includes sarcopenia and some of its consequences and is a powerful predictor of mortality [9, 10]. Adipose tissue gain also is very common in aging and is a growing health concern for all ages [11, 12]. Visceral (abdominal) fat is of the greatest health concern because it is associated with insulin resistance, type 2 diabetes, cardiovascular disease, dementia, cancer, and overall mortality [3, 13]. Although obesity can be associated with normal, increased, or decreased muscle mass, muscle weight and even muscle fiber size measurements can be misleading in aging because muscle becomes infiltrated with fat [5, 14]. Furthermore, obesity prevents muscle gain in response to functional overload, possibly through skeletal muscle mTOR hyperactivation [15, 16]. Importantly, the combination of obesity and sarcopenia (so-called sarcopenic obesity) carries high health risks [5].

Another hallmark of aging is declining adaptive immunity, with complex alterations in innate immunity [17-19]. Immune senescence is associated with mortality from all causes, including infectious diseases [17, 20]. Natural killer (NK) lymphocytes are innate immune cells that control intracellular infectious agents and cancers. In contrast to T and B lymphocytes, NK cell number is relatively increased in healthy aging and defects in NK cell function are subtle [21, 22]. However, declining NK cell number or function in aging is associated with death in the elderly [23, 24]. Therefore, mechanisms that preserve NK cell number and function may promote healthy aging.

**Figure 1:**
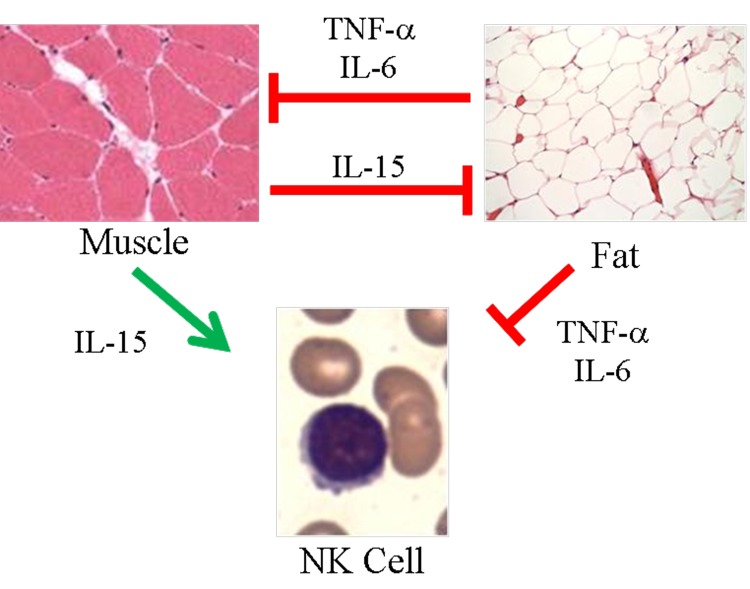
Proposed model of skeletal muscle, adipose tissue, and NK cells in aging Muscle negatively regulates adipose tissue via IL-15 and possibly other myokines. In turn, inflammatory adipokines produced by adipose tissue, especially visceral fat, negatively regulate muscle. These same myokines and adipokines are proposed to affect NK cells. IL-15 is required for NK cell development and survival, whereas inflammatory cytokines such as TNF-α and IL-6 shorten NK cell survival or stimulate NK cells to produce pro-inflammatory IL-17. During aging, muscle mass, strength, and endurance decline, which we propose results in less IL-15 production. At the same time, increased adipose tissue that is characteristic of aging is associated with increased inflammatory adipokine production. We propose that these changing myokines and adipokine levels negatively affect NK cells during aging.

**Figure 2:**
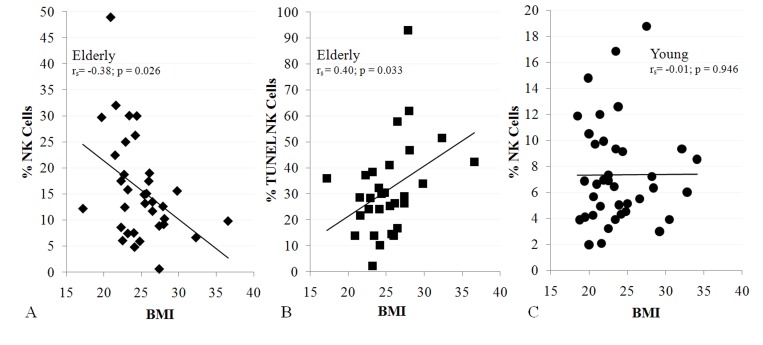
NK cells are related to BMI in the elderly (**A**, 34 subjects; **B**, 30 subjects), but not in the young (**C**, 38 subjects). Shown is BMI correlation with% NK cells (**A**, **C**) and NK cell apoptosis (**B**). All subjects were healthy, non-diabetic, and had BMI < 37. Least squares lines, Spearman correlation coefficient (r_s_) and significance are noted. Investigation has been conducted in accordance with the ethical standards and according to the Declaration of Helsinki and according to national and international guidelines and has been approved by the University of Kentucky human subjects review board.

To relate sarcopenia, obesity, and declining immunity in aging, we speculated that these conditions are linked processes, which are controlled by adipose tissue-derived and skeletal muscle-derived cytokines, known as adipokines and myokines, respectively (Figure 1). As a preliminary exploration of this potential relationship, we studied body mass index (BMI) and NK cells in healthy young (median age 27) and elderly (median age >80) women. In the elderly cohort, BMI correlated inversely with percentage NK cells and correlated directly with NK cell apoptosis rate (Figure 2A, B). These relationships were not seen in young women (Figure 2C & not shown). As is typical for this age group, elderly subjects had a relatively high percentage NK cells among blood mononuclear cells, which is due both to higher NK cell number and to lower numbers of other blood lymphocytes. This may be a protective mechanism in the elderly when other leukocyte functions decline [17-19]. Our data suggest that the relative increase in NK cells with aging is blunted by obesity.

We propose that the link between body composition (the increased ratio of fat to muscle) and immunity in aging is due to increased expression of adipokines and decreased expression of myokines that affect the immune system. In order to explore this proposed link (Figure 1), we will review selected adipokines and myokines. Many recent reviews have described adipokine and myokine effects on muscle, fat, and metabolism [3, 25-28], so we will concentrate on how adipokines and myokines affect the immune system with special reference to NK cells. Starting with adipokines, our review indicates that as adipose tissue mass increases, the amount of the anti-inflammatory cytokine, adiponectin, falls. At the same time, there is a rise in the amount of the pro-inflammatory molecules leptin, chemerin, resistin, TNF-α, IL-1, and IL-6. These proinflammatory adipokines have complex effects on NK cells. Therefore, obesity is associated with a strongly pro-inflammatory state that promotes sarcopenia and may inhibit NK lymphocytes.

### Adipose Tissue Cytokines—Adipokines

Adipokine-producing cells include adipocytes themselves, as well as adipose tissue leukocytes. The spectrum of secreted adipokines varies with diet (high and low fat), energy balance (obese and lean), and adipose tissue location (visceral and peripheral). Much attention has been focused on adipose tissue macrophages, which secrete considerable quantities of cytokines. In both mice and humans, high fat diet and obesity are associated with increased adipose tissue macrophages, which are responsible for secreting most of the IL-6 and TNF-α produced by adipose tissue [29]. T and NK lymphocytes also reside in adipose tissue [30-33], but how they shape adipose tissue macrophage activation is not completely understood. In both mice and humans, NK cell numbers are higher in visceral fat than in other fat depots [31, 32]. Fat-associated NK cells have been reported to increase or decrease with high fat diet in rodents, depending upon the fat depot examined and the cell surface markers used to identify NK cells [30, 31, 34]. Knockout of the interferon (IFN)-γ gene led to a decrease in fat-associated NK1.1^+^ cells (mostly NK cells) [34]. Given that NK cells are major IFN-γ producers, this suggests a potential regulatory loop between NK cells and other fat-associated leukocytes. In humans, CD56^+^ cells (mostly NK cells) were more numerous in visceral fat from obese subjects than from lean subjects and were more numerous in visceral fat than subcutaneous fat [35]. Although the exact role of NK cells in adipose tissue has not been defined, they are present and are likely to be important.

Primarily a product of adipocytes, adiponectin declines with increasing fat mass [27, 28]. Adiponectin also declines with age, although some or all of this may be due to increasing fat mass with age [36]. Adiponectin activates AMP-activated protein kinase and inhibits NF-κB signaling, decreasing monocyte, macrophage, and dendritic cell production of TNF-α and IFN-γ, while increasing anti-inflammatory cytokines, IL-10 and IL-1Ra [27, 28]. Adiponectin directly inhibits natural killer (NK) cells by preventing IL-2-stimulated cytotoxicity and IFN-γ production [37].

In contrast to adiponectin, serum leptin levels reflect overall adipose mass [27]. Leptin increases TNF-α, IL-6, and IL-12 production by monocytes [27, 38]. Therefore, leptin is largely proinflammatory. Leptin receptor-deficient (*db/db*) mice have low NK cell numbers [39]. However, no statistically significant associations were observed between serum leptin and human NK cytotoxicity, lymphocyte populations (including NK, CD4 or CD8 T cells, or CD19 B cells), or overall T cell proliferation [40]. These findings may be related to leptin resistance in obese humans [41], because human NK cells respond to short-term leptin stimulation in vitro, but not to long-term leptin stimulation [42]. Furthermore, NK cells from lean rats, but not high fat diet-induced obese rats, showed significantly increased cytotoxic activity 4 hours after leptin injection [43]. Leptin resistance appears to increase in aging rats, even without obesity [44].

The leukocyte chemotactic factor, chemerin, is produced from the Tazarotene-induced gene 2 as an inactive precursor, prochemerin, by many tissues, with liver and adipose tissues as major sources [45, 46]. Prochemerin is processed by inflammatory, coagulation, and fibrinolytic proteolytic enzymes into the active chemokine or into inactive products, indicating complex regulation. NK cells and most other leukocytes express the ChemR23 G-coupled receptor for chemerin and migrate toward higher concentrations [45]. Chemerin, which increases with obesity [46], may be responsible for the NK cell, macrophage, and other leukocyte recruitment to adipose tissue. Interestingly, adipocytes express both chemerin and ChemR23 and the resulting autocrine signaling may contribute to adipocyte differentiation [45]. Adipocyte chemerin synthesis is elevated by both TNF-α and IL-1β, suggesting a possible feed-forward loop of leukocyte recruitment and inflammatory cytokine production, leading to greater leukocyte recruitment [45]. The effects of aging on chemerin production are not known.

Resistin is made by adipocytes, muscle cells, and leukocytes [27]. Resistin induces TNF-α and IL-6 secretion by human blood leukocytes and, in turn, resistin expression is induced by IL-1, TNF-α, and IL-6 [27, 47], implying a potential feed-forward loop of proinflammatory cytokines. Resistin RNA is expressed at higher levels in visceral fat than in non-abdominal subcutaneous fat [48] and serum resistin levels are higher in obese subjects than in lean subjects [49]. Resistin does not change with age in healthy subjects [36]. To our knowledge, the effects of resistin on NK cells, if any, have not been characterized.

Extracellular nicotinamide phosphoribosyltransferase (Nampt; pre-B-cell colony enhancing factor, PBEF; visfatin) upregulates proinflammatory cytokines in monocytes, including IL-1β, TNF-α, and IL-6 [28]. Nampt also induces monocyte cell surface ligands for stimulatory lymphocyte receptors, including CD80 (B7-1), CD40, and CD54 (ICAM-1), increases macrophage phagocytosis, and prevents neutrophil apoptosis [28, 50]. Although it had been touted as a product of visceral adipose tissue, Nampt is produced by many tissues, including skeletal muscle [51-53]. Nampt measurement is sensitive to assay variation and it is not clear whether blood Nampt levels correlate with fat mass [54]. To our knowledge, the effects of Nampt on NK cells and the effects of aging on extracellular Nampt production have not been studied.

Tumor necrosis factor-α (TNF-α) is a powerful proinflammatory cytokine that is made primarily by monocyte, macrophages, and similar cells, such as liver Kupffer cells and brain microglia. TNF-α also is made by other cells including, B, T, and NK lymphocytes, and by adipocytes [55]. TNF-α also is made by human skeletal muscle, especially from diabetic subjects, and when cells are stimulated with proinflammatory cytokines, including TNF-α itself [56, 57]. In visceral adipose tissue, TNF-α is made predominantly by adipose tissue macrophages [29, 32, 58]. The elevated systemic TNF-α that is strongly associated with obesity comes largely from visceral adipose tissue [29, 59]. TNF-α increases with age, even among healthy subjects [60, 61]. Although largely proinflammatory, TNF-α causes T cell and NK cell apoptosis under some circumstances [62-64].

IL-6 is produced by many cells, including adipocytes, adipose tissue macrophages, and myocytes [28, 29]. IL-6 levels rise with obesity and with age [29, 60, 61, 65-67]. Chronic IL-6 elevation might have different effects than the transient elevations that are found after exercise [68]. IL-6 has many effects on the immune system, which are generally pro-inflammatory, including promotion of pro-inflammatory T_H_17 cells, IL-17-producing NK cells, and suppression of regulatory T cells [69, 70]. However, IL-6 may be anti-inflammatory in some circumstances in humans because it inhibits TNF-α release, both basally and in response to endotoxin infusion, and it induces release of anti-inflammatory molecules, IL-1Ra, IL-10, and cortisol [71, 72].

Proinflammatory IL-1β and IL-1α are produced by many cells, including adipocytes and myocytes, but most prominently by monocytes and macrophages. The IL-1 receptor-blocking protein, IL-1Ra (anakinra), has been successfully used to treat many autoinflammatory diseases, providing strong evidence for the pro-inflammatory nature of IL-1β and IL-1a [73]. In obesity, fat tissue is a source of IL-1 [74] and human IL-1β rises with age [61]. IL-1β supported a unique IL-22^+^ subpopulation of secondary lymphoid tissue “stage 3” immature NK cells and promoted the proliferation of a specialized class of IL-22+ NK-22 mucosal cells [75, 76]. For conventional NK cells, the picture was mixed—IL-1β inhibited development of conventional NK cells, but with IL-12 co-stimulated immature CD56^bright^ NK cells to produce IFN-γ [75, 77].

Serum IL-10 may be positively correlated with obesity in middle aged humans [78]. Exercise releases IL-10 into the circulation, implying production by skeletal muscle [3]. Macrophage IL-10 production increases in old mice [79, 80]. However, serum IL-10 levels are quite variable in elderly humans. One study showed a significant age-related increase in serum IL-10, whereas an earlier study did not show a significant difference between middle-aged and very old humans [61, 67]. IL-10 is broadly anti-inflammatory, inhibiting antigen presentation and suppressing release of TNF-α, IL-2, IFN-γ, IL-4, and other cytokines [81]. The immunoregulatory nature of IL-10 is illustrated by the fact that several viruses encode IL-10 homologues [81]. The effect of IL-10 on NK cells is variable, depending upon context. IL-10 stimulated NK cell proliferation, cytotoxicity, and cytokine secretion in vitro when combined with IL-2 [82]. In murine cytomegalovirus-infected mice, IL-10 promoted NK cell cytotoxic granule release, but increased NK cell activation-induced cell death [83].

Transforming growth factor-β (TGF-β) is produced by many cells, including adipose tissue macrophages in insulin resistance and NK cells in both constitutive and activation-dependent fashions [84, 85]. Although total serum TGF-β levels do not change with age, active TGF-β levels do increase [67]. TGF-β inhibits NK cell cytotoxicity, IFN-γ production, and homeostasis, by downregulating important NK cell receptors, including the IFN-α receptor, IL-2Ra, NKp30, and NKG2D [84]. Therefore, adipose-derived TGF-β might negatively regulate NK cells.

In summary, as adipose tissue mass increases, the amount of anti-inflammatory adiponectin decreases and the amounts of the pro-inflammatory molecules leptin, chemerin, resistin, TNF-α, IL-1, and IL-6 increase. Obesity is associated with a strongly pro-inflammatory state that has complex effects on NK lymphocytes. Because of these complicated relationships, it is not surprising that obesity per se did not correlate with NK cell number or activity in several studies [86-88]. However, obese patients with metabolic syndrome or components of metabolic syndrome had low NK cell number and/or activity. For example, when Lynch et al. [88] separated obese subjects with and without components of the metabolic syndrome, those with metabolic syndrome components had significantly lower NK cell number and less NK cell activation than obese subjects without metabolic syndrome components. In a study of obese patients undergoing bariatric surgery, patients had lost weight and improved many components associated with the metabolic syndrome 6 months after surgery [89]. Although there was no change in CD56^+^ cell number (which would include NK cells and some T cells), NK cell activity greatly increased after surgery [89]. Given that low muscle mass and strength are a frequent in patients with metabolic syndrome [90, 91], this suggested that sarcopenia could synergize with excess adipose tissue to affect NK cells, as was implied by our preliminary study (Figure 2).

Below we review myokines that have been shown to affect NK cells, with special emphasis on IL-15. Declining muscle mass is expected to produce less IL-15, a myokine that regulates adipose tissue and promotes NK cell development and survival. Therefore, sarcopenia is expected to hinder NK cells.

### Skeletal Muscle Cytokines—Myokines

Skeletal muscle secretes multiple cytokines, including Nampt, TNF-a, and IL-6, which were discussed above. Muscle also produces IL-8, brain-derived neurotrophic factor, and leukemia inhibitory factor, which are not secreted into the circulation in large amounts, and fibroblast growth factor-21, which has unknown effects on the immune system. Insulin-like growth factor-I (IGF-I) is made by liver, skeletal muscle, monocytes, and other cells. IGF-I administration enhances mouse lymphoid and myeloid reconstitution after allogeneic bone marrow transplantation [92]. The relatively high level of type-1 IGF receptors on NK cells suggests that IGF-I is likely to affect NK cells [93], but this has not been documented.

IL-15 mRNA is expressed in many tissues [94], but IL-15 biosynthesis is very complex and RNA levels do not necessarily indicate protein secretion. IL-15 isoforms have alternative signal peptides of 21 and 48 amino acids. Although the shorter form is more conventional in length, it has a noncanonical Kozak translational initiation sequence and is poorly expressed [95]. The IL-15 long signal peptide isoform also has several features that cause poor protein expression, including an abnormal length and the presence of 12 “decoy” AUGs in the 5'UTR. IL-15 mRNA also has poorly defined inhibitory elements in the 3'UTR. These features partially account for low IL-15 protein expression despite abundant RNA in many cells.

Importantly, IL-15 requires the presence of IL-15Rα for efficient biosynthesis and secretion [96, 97]. IL-15 and IL-15Rα have high affinity (~10^−10^ K_d_), bind within the cell, are transported to the cell membrane, and are shed in a process that involves the matrix metalloproteinase, ADAM17 [98-100]. Another secretory route has been proposed in which alternative RNA splicing produces a soluble IL-15Rα (sIL-15Rα) protein that is mostly limited to the IL-15-binding “sushi” domain. Although recombinant “sushi-only” sIL-15Rα has somewhat reduced affinity for IL-15 compared with shed sIL-15Rα, soluble “sushi-only” complexes might be secreted [100]. Either spliced “sushi-only” or shed sIL-15Rα is absolutely required for IL-15 secretion. Because of their high affinity, sIL-15Rα/IL-15 complexes have a long (12 hour) half-life [100]. However, the total serum IL-15 concentration is low, perhaps ~6-20 pg/ml (~10^−12^ M) in healthy subjects [101, 102], favoring complex dissociation. Human “free” (non-complexed) IL-15 had a circulating half-life of only about 1 hour when injected into mice [103]. This is expected because, at 14-15 kDa mass, IL-15 probably is rapidly filtered by the kidneys [104]. As a result, most or all of IL-15 is bound to IL-15Rα in human and mouse serum, at least when IL-15 levels are elevated by lymphocyte-depleting treatments [105].

Like IL-15, IL-15Rα synthesis is widespread within and outside of lymphoid tissues. Skeletal muscle tissue produces very high levels of IL-15, and expresses IL-15Rα [106]. Because skeletal muscle is the largest organ system in the body, these observations suggest that skeletal muscle could be an important IL-15 producer with endocrine effects at distant sites. IL-15 levels are reported to increase transiently immediately following resistance [107] and aerobic [108] exercise, suggesting that IL-15 is indeed released from muscle tissue. In mice, muscle and serum IL-15 protein levels decline progressively with advanced age [109]. In agreement with the above-mentioned lack of correlation between IL-15 protein levels and mRNA expression, the age-related decline in muscle IL-15 protein was not associated with decreases in IL-15 mRNA levels [109]. However, an age-related decline in expression of mRNA coding for the “sushi-only” isoform of sIL-15Rα was observed, suggesting the reduction in this factor resulted in reduced IL-15 secretion and stability in aging mice [109]. A study of aging rats showed the longevity-promoting regimen of calorie restriction prevented the age-related declines in muscle IL-15 expression observed in ad libitum-fed rats [110]. IL-15 is reported to prevent muscle protein degradation in certain model systems [111-113]. In an intriguing brief report involving human subjects, Gangemi et al. [114] observed significantly elevated serum IL-15 levels in centenarians living independently, suggesting high expression of IL-15 conferred protection from both frailty and age-related disease.

IL-15 also has important effects on adipose tissue. IL-15 inhibits adipocyte differentiation in culture and obese people have low blood IL-15 levels [102, 106, 115]. IL-15 deficient mice become obese despite unaltered food consumption; IL-15 injection reversed both this obesity and diet-induced obesity, lowered glucose levels, and increased insulin sensitivity [102, 116]. Consistent with these findings, high IL-15 levels caused lean body type, decreased white adipose tissue, and increased adipose tissue NK cell number [102, 112, 117]. These and other data support the conclusion that IL-15 regulates adipose tissue [3, 106, 115].

IL-15 is best known for its effect on the immune system. NK cells and select T cell subsets express three chains important for IL-15 signaling: a signal-transducing γ chain (CD132) that is used by several cytokine receptors, an IL-15-binding β chain (CD122) that is important for both IL-15 and IL-2 signaling and is often referred to as the IL-2Rβ chain, and an α chain (IL-15Rα) that, as noted above, binds IL-15 with high affinity [95, 100]. Complexes of these three chains on lymphocytes are referred to as IL-15Rabg. NK cells absolutely require IL-15, as IL-15 and IL-15Rα knockout mice have virtually no mature NK cells and normal NK cells rapidly die when transferred into these mice [118-121]. IL-15 elevates bcl-2 and protects NK cells from apoptosis [122, 123]. Two observations indicate that physiological IL-15 levels are rate-limiting for NK cells: heterozygous IL-15 knockout mice have low NK cell number and exogenous IL-15 boosts NK cell number in both normal mice and primates [119, 124, 125]. Specific T cell subsets also depend upon IL-15, including CD8 memory cells, NKT cells, and some intraepithelial T cells [118, 119, 126].

In addition to being shed, IL-15Rα/IL-15 complexes can be stably expressed on cell membranes. Both soluble and cell surface IL-15Rα/IL-15 complexes prolong IL-15 stability and effectively stimulate NK cells and T cells, a process called transpresentation [96, 97, 127, 128]. Multiple lines of evidence indicate that NK cells and certain T cell subsets require IL-15Rα expression by other cells, but need not express it themselves [127, 128]. Several groups have investigated which cells are critical for IL-15 transpresentation in vivo. In adoptive transfer experiments, IL-15Rα expression by both radiosensitive hematopoietic cells and radioresistant stromal cells were required for optimal lymphocyte maturation [126, 128]. In particular, peripheral NK cells were partially supported by IL-15Rα/IL-15 expression by either hematopoietic or nonhematopoietic cells, but both compartments were required for optimal NK cell number [121, 126, 128]. Mortier et al. selectively knocked out IL-15Rα expression within the hematopoietic compartment. Knockout of IL-15Rα in either dendritic cells or macrophages produced partial loss of NK cells in peripheral organs, but not in the bone marrow [129]. Curiously, loss of IL-15Rα expression in *both* dendritic cells and macrophages produced similar results to that of either knockout separately [129]. Furthermore, survival of normal transferred NK cells into either knockout animal was normal. Together, these results indicate that nonhematopoietic cells contribute significantly to NK cell maturation and survival.

Because IL-15Rα is required for IL-15 biosynthesis, stability, and transpresentation, it is relevant to consider the role of IL-15Rα in NK and T cells, which do not produce IL-15. Logically, if transpresentation is both necessary and sufficient for IL-15 signaling, then there would be no need for IL-15Rα expression on responding NK and T lymphocytes. However, considerable data indicate that lymphocyte IL-15Rα is required for optimal response to IL-15. As a first consideration, it is clear that IL-15Rabg+ lymphocytes can respond to free IL-15. Cell lines expressing human IL-15Rabg responded with exquisite sensitivity to human IL-15 (about 10 pM) [130]. sIL-15Rα may prevent free IL-15 from binding to lymphocyte IL-15Rα because these cell lines were inhibited from growing in response to IL-15 when sIL-15Rα was included, with an IC50 of 3-10 pM [98, 130]. Similarly, a human T cell line was rescued from cell death by IL-15, but this was abolished when IL-15Rα was included in the medium [131]. IL-15Rα also inhibited allogeneic responses in vivo [131]. Lymphocytes with only IL-15Rbg bind IL-15 with approximately 700-fold lower affinity than those with IL-15Rabg [132]. Mouse CTLL T cells responded well to free IL-15, but CTLL variants that expressed little IL-15Rα did not [130, 131]. The presence of sIL-15Rα inhibited the proliferative response to IL-15 by CTLL, but enhanced the proliferation by IL-15Rα-low CTLL cells [130, 131]. The effect of sIL-15Rα on lymphocyte response to free IL-15 may depend upon the ratio of “complete” IL-15Rabg to “incomplete” IL-15Rbg on the lymphocyte cell surface. Collectively, these data indicate that IL-15Rabg+ lymphocytes respond with exquisite sensitivity to free IL-15.

NK and T lymphocytes express IL-15Rbg, allowing them to respond to sIL-15Rα/IL-15 complexes, in addition to IL-15Rα/IL-15 transpresented on cell surfaces. The ratio of IL-15Rabg to IL-15Rbg likely depends upon the state of cell activation. Free IL-15 stimulated resting human NK cells, but IL-15Rα expression was transient and disappeared by 48 hours, possibly due to ADAM17-induced cleavage from the NK cell surface [133]. However, activated NK cells still responded to sIL-15Rα/IL-15 complexes [133]. Thus, NK cells require IL-15Rα to respond to “free” IL-15 and NK cells that have low cell surface IL-15Rα can respond to transpresented IL-15Rα/IL-15 complexes that are soluble or that are on the surface of neighboring cells.

It is important to address apparent species and cell line differences that complicate the IL-15 literature. For mouse lymphocytes, “full length” shed IL-15Rα inhibits signaling when complexed with IL-15 [130, 131]. This also is true for transfectants that may express high levels of human IL-15Rα [98]. However, for human blood lymphocytes, “full length” sIL-15Rα/IL-15 complexes either further stimulate or have only a minor inhibitory effect, compared with IL-15 alone [99, 130, 134]. Thus sIL-15Rα/IL-15 complexes, whether derived from “shed” or “spliced” IL-15Rα, likely stimulate human IL-15Rbg^+^ NK lymphocytes in vivo.

### Hypothesis

As noted above, physiological IL-15 levels are rate-limiting for NK cells. This implies that any source of IL-15 will boost NK cell number and function. As also noted above, knockout studies indicate that non-hematopoietic cells play an important role in NK homeostasis. Although cell surface transpresentation is likely to efficiently deliver IL-15 proliferation and survival signals to NK cells and T cells in a juxtacrine fashion, we propose that soluble IL-15Rα/IL-15 complexes and free IL-15 provide a small but significant endocrine effect. Given the size of the skeletal muscle tissue and its abundant production of IL-15 and IL-15Rα, we propose that skeletal muscle contributes to NK cell homeostasis. As a corollary, we propose that muscle wasting results in lower available sIL-15Rα/IL-15 complex and free IL-15 levels, leading to less robust NK cell homeostasis. When muscle wasting is combined with elevated inflammatory cytokines produced by adipose tissue, some of which negatively affect NK cells, we predict that this lowers NK cell number and survival, much as we saw in elderly female subjects with high BMI. Relevant to this concept, chronic diseases of the lung, heart, and kidney are characterized by muscle wasting and low NK cell numbers [135-138]. Obesity, loss of muscle, and low NK cell number all predict mortality in the elderly [23, 24, 139-141], suggesting that these factors might combine to promote diseases of the elderly, including infection and cancer.
